# CD226 reduces endothelial cell glucose uptake under hyperglycemic conditions with inflammation in type 2 diabetes mellitus

**DOI:** 10.18632/oncotarget.7505

**Published:** 2016-02-19

**Authors:** Yuan Zhang, Tian Liu, Yu Chen, Zilong Dong, Jinxue Zhang, Yizheng Sun, Boquan Jin, Feng Gao, Shuzhong Guo, Ran Zhuang

**Affiliations:** ^1^ Department of Aerospace Medicine, Fourth Military Medical University, Xi'an, China; ^2^ Department of Plastic Surgery, Xijing Hospital, Fourth Military Medical University, Xi'an, China; ^3^ Department of Gastroenterology, Xijing Hospital, Fourth Military Medical University, Xi'an, China; ^4^ Department of Dermatology, Xijing Hospital, Fourth Military Medical University, Xi'an, China; ^5^ Department of Immunology, Fourth Military Medical University Xi'an, China

**Keywords:** CD226, high fat diet, type 2 diabetes mellitus, endothelial cells, Pathology Section

## Abstract

CD226 is a co-stimulatory adhesion molecule found on immune and endothelial cells. Here, we evaluated a possible role for CD226 in inhibiting glucose uptake in isolated human umbilical vein endothelial cells (HUVECs) and in wild-type (WT) and CD226 knockout (KO) mice with high-fat diet (HFD)-induced type 2 diabetes (T2DM). CD226 expression increased under hyperglycemic conditions in the presence of TNF-α. Furthermore, CD226 knockdown improved glucose uptake in endothelial cells, and CD226 KO mice exhibited increased glucose tolerance. Levels of soluble CD226 in plasma were higher in T2DM patients following an oral glucose tolerance test (OGTT) than under fasting conditions. Our results indicate that low-grade inflammation coupled with elevated blood glucose increases CD226 expression, resulting in decreased endothelial cell glucose uptake in T2DM.

## INTRODUCTION

Type 2 diabetes mellitus (T2DM), which affects more than 150 million people worldwide, increases the risk of cardiovascular mortality [1, 2]. Chronic low-grade inflammation in T2DM patients orchestrates obesity-induced insulin resistance [3]. Diabetic hyperglycemia causes a variety of pathological changes in blood vessels. Blood vessel endothelial dysfunction is characterized by impaired vasodilation, altered vascular remodeling, and loss of barrier function, which leads to increased permeability [4]. Some studies show that insulin delivery to the skeletal muscle interstitium through endothelial cells is the rate-limiting step in insulin-stimulated glucose uptake [5]. However, the molecular mechanisms by which hyperglycemia leads to vascular diseases in diabetics remain unknown.

CD226, also known as DNAX accessory molecule 1 (DNAM-1) and platelet and T cell activation antigen 1 (PTA-1), is a type I transmembrane glycoprotein expressed primarily on the surface of NK cells, T cells, and platelets. CD226 binds to CD155/CD112 and activates intracellular signaling cascades that lead to cell-specific responses, including immune cell activation and target cell lysis. Thus, CD226 appears to be an integral component of the immune response in cancer, allergic inflammatory disorders, and autoimmune diseases [6-10]. CD8^+^ T cells require CD226 to recognize antigens presented by non-professional antigen-presenting cells. Similarly, NK cells require CD226 for elimination of tumor cells that are resistant to cytotoxicity [11]. We previously showed that CD226 is expressed very weakly in resting human umbilical vein endothelial cells (HUVECs), but CD226 levels increased when these cells were stimulated [12].

## RESULTS

### Endothelial cells expressed CD226

**Figure 1:**
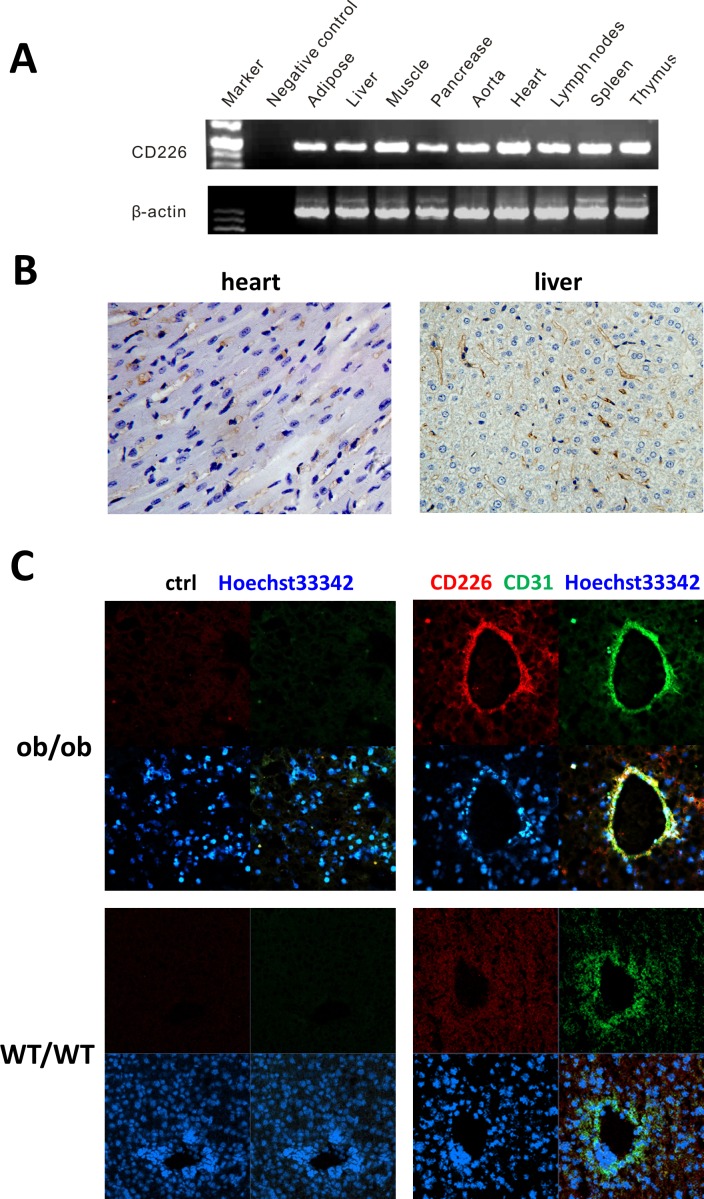
CD226 distribution in WT C57BL/6 mouse endothelial tissues **A.** RT-PCR analysis. CD226 was expressed in immune tissues such as the lymph nodes, spleen, and thymus, in tissues involved in glucose metabolism, and in the cardiovascular system. The data shown represent 3 independent experiments. β-actin was the RT-PCR internal control. Negative control reactions were conducted without cDNA. **B.** Immunohistochemistry (IHC) staining of CD226 in normal mouse heart and liver. CD226 was detected in endothelial cells (original magnification, ×400), *n* = 3-5 for each group of mice. **C.** CD226 expression in liver sinusoidal endothelial cells, marked by CD31 staining, was higher in ob/ob mice (upper) than in WT mice (lower).

RT-PCR was used to assess tissue distribution of CD226 in wild-type C57BL/6 mice (Figure 1A). Transcripts of about 200 bp, corresponding to the size predicted by the mouse CD226-specific primers, were detected in the lymph nodes, spleen, thymus, and tissues related to glucose metabolism (adipose, liver, muscle, and pancreas). In addition, the heart, aorta, and immune tissues also expressed CD226 transcripts.

CD226 immunohistochemistry staining was performed to explore CD226 expression in different cell types in the heart and liver. CD226 was mainly found in vascular endothelial cells in the heart and sinusoidal endothelial cells in the liver (Figure 1B). Vascular endothelial cells line the entire circulatory system, which might explain why CD226 was also expressed broadly in the cardiovascular system and glucose metabolic tissues. In addition, CD226 expression in liver sinusoidal endothelial cells marked by CD31 (green) was higher in ob/ob mice than in WT mice (Figure 1C).

### High glucose levels together with TNF-α increased endothelial cell CD226 levels

**Figure 2:**
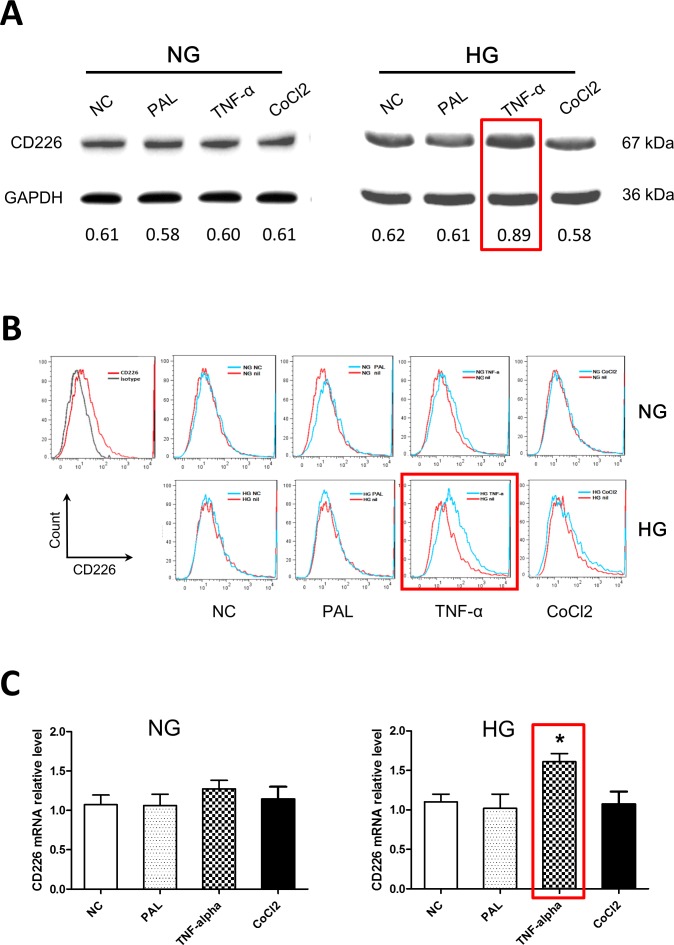
High glucose and TNF-α treatment increased CD226 expression in HUVECs Confluent HUVECs were exposed to 5.5 mM (normal glucose, NG) or 30 mM glucose (high glucose, HG), with or without 200 μM palmitate, 10 ng/ml TNF-α, or 200 μM CoCl_2_ for 12 h in endothelial cell growth medium-2. Representative Western blot analysis of changes in CD226 compared to negative control (without stimulation) in normal glucose (**A.**, left) and high glucose (**A.**, right) cells. GAPDH served as an internal control. Relative expression levels are shown at the bottom. Increased CD226 was also detected by flow cytometry staining **B.** and qPCR at the mRNA level **C.**. Data are shown as the mean of three independent experiments. * *p* < 0.05 compared to the negative control group.

HUVECs were exposed to 5.5 mM or 30 mM glucose for 12 h in the presence or absence of palmitate, TNF-α, or CoCl_2_. Palmitate is a typical saturated free fatty acid (FFA), TNF-α is a key inflammatory factor, and CoCl_2_ is a chemical hypoxia reagent. CD226 expression did not differ initially between the normal and high glucose groups of untreated endothelial cells (negative control groups). Under the normal glucose condition (5.5 mM), palmitate, TNF-α, and CoCl_2_ had no obvious effects on CD226 expression. However, TNF-α increased CD226 expression in the high glucose condition (30 mM, *p* < 0.05); palmitate and CoCl_2_ still had no effect (Figure 2A). We also measured the increase in CD226 levels by flow cytometry staining and qPCR at the protein and mRNA levels, respectively (Figure 2B, 2C). These results indicate that CD226 levels in endothelial cells may increase in response to low-grade inflammation under hyperglycemic conditions. It is unclear whether this is a protective mechanism or a biological marker of endothelial cell dysfunction.

### CD226 knockdown increased endothelial cell glucose uptake under high glucose conditions with inflammation

**Figure 3:**
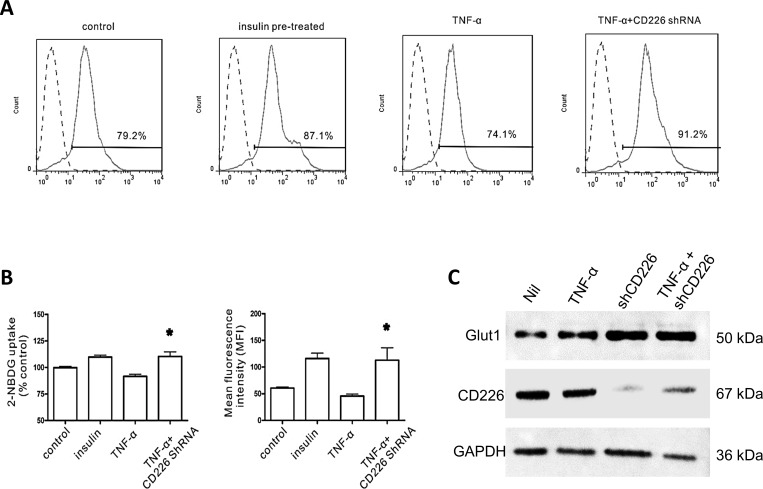
Effect of CD226 on 2-NBDG uptake in HUVECs Cells were pre-incubated with 100 μM 2-NBDG for 30 min. Flow cytometry histogram of 20,000 cells. **A.** Representative flow cytometry analysis of 2-NBDG glucose uptake following different treatments. **B.** Glucose uptake expressed as percent of control and mean changes in 2-NBDG fluorescence intensity (MFI). Significant differences *vs*. the TNF-α group are indicated, * *p* < 0.05. **C.** Membrane expression of Glut1 increased after CD226 shRNA lentivirus infection with or without TNF-α treatment. Data are shown as the mean of three independent experiments.

The 2-NBDG glucose uptake assay is a sensitive and non-radioactive method for directly and rapidly measuring glucose uptake in single, living cells [13]. We found that optimal staining was obtained following incubation with 100 μM 2-NBDG at 37°C for 30 min. 10^−7^M insulin increased 2-NBDG uptake in HUVECs by 9.8±1.8% compared to the control group. The mean fluorescence intensity (MFI) of the insulin group was 116.0±10.0 compared to 60.8±1.8 in the control group. TNF-α alone decreased glucose uptake by 8.2±3.2%, and the resulting MFI was 45.5±3.9. CD226 knockdown increased 2-NBDG uptake by 10.6±3.1% in the presence of TNF-α, and the associated MFI was 112.9±23.1 (*p* < 0.05 *vs*. TNF-α group, Figure 3A and 3B). Glucose uptake was unaffected in all experiments in which cells were exposed to high glucose and treated with CD226 shRNA lentivirus alone without TNF-α (data not shown). Furthermore, CD226 shRNA lentivirus infection increased membrane expression of the primary glucose transporter Glut1 whether or not TNF-α was present (Figure 3C). Taken together, these results indicate that CD226 expression increases during high glucose conditions with low-grade inflammation. Conversely, CD226 knockdown increases endothelial cell glucose uptake.

### CD226 knockdown decreased high glucose- and inflammation-induced cytoskeleton remodeling in endothelial cells

**Figure 4:**
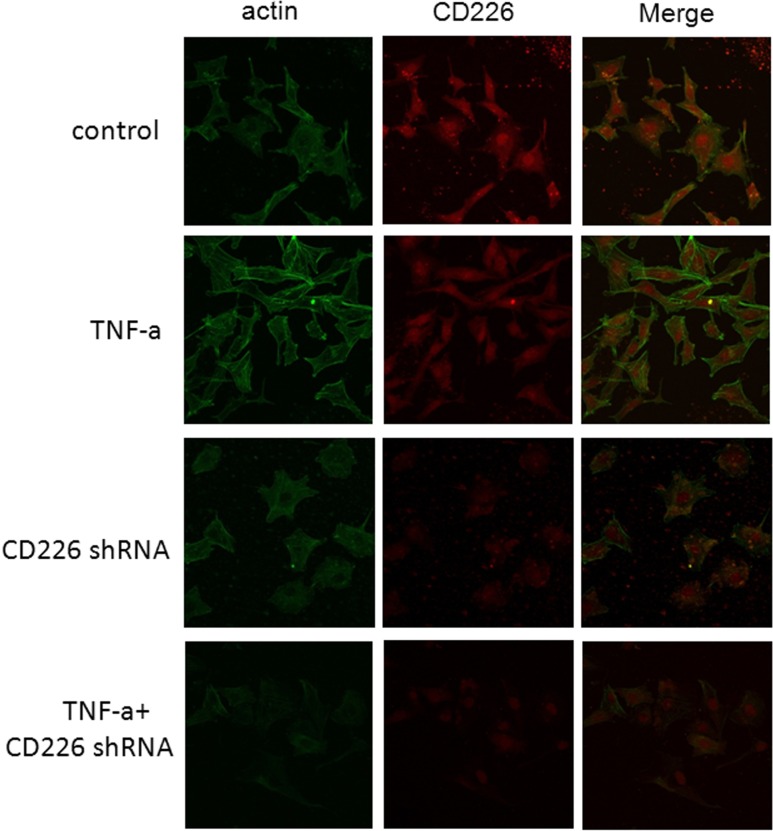
CD226 knockdown reduced inflammation-induced cytoskeleton remodeling in endothelial cells under high glucose conditions HUVECs grown on chamber-slides after treatment were fixed and stained with FITC-phalloidin to detect F-actin as described in Materials and Methods. **A.** Control cells pretreated with scrambled control shRNA for 48 h and high glucose for another 12 h had few stress fibers. **B.** Following incubation with 10 ng/ml TNF-α and high glucose for another 12 h, F-actin was redistributed to the subcortical compartment and stress fibers formed. **C.**, **D.** Pretreatment with CD226 shRNA lentivirus for 48 h prevented redistribution of F-actin with or without TNF-α stimulation and 12 h of high glucose. Results are representative of three independent experiments.

Both high glucose and TNF-α impair endothelial barrier function and increase vascular permeability [14]. Rearrangement of filamentous actin (F-actin), the predominant cytoplasmic microfilament in non-muscle cells, is thought to be responsible for this altered permeability in HUVECs [4]. In morphologic studies using fluorescent staining of the actin cytoskeleton, we found that few stress fibers formed after 12 h exposure to high glucose. Stress fiber formation increased and F-actin was redistributed to the subcortical compartment after TNF-α was added. Pretreatment with human CD226 shRNA lentivirus reduced the redistribution of F-actin and formation of stress fibers under high glucose conditions both with and without 12 h of exposure to TNF-α (Figure 4).

### CD226 knockout reduced HFD-induced glucose intolerance in mice

To determine the physiologic role of CD226 in non-immune tissues, we analyzed genes related to glucose metabolism with the RT^2^Profiler^TM^ PCR Array. After 14 weeks on a high fat diet (HFD), no differences were detected between the livers of WT mice and CD226 KO mice (Supplemental Table 1 and Supplemental Figure 1).

**Figure 5:**
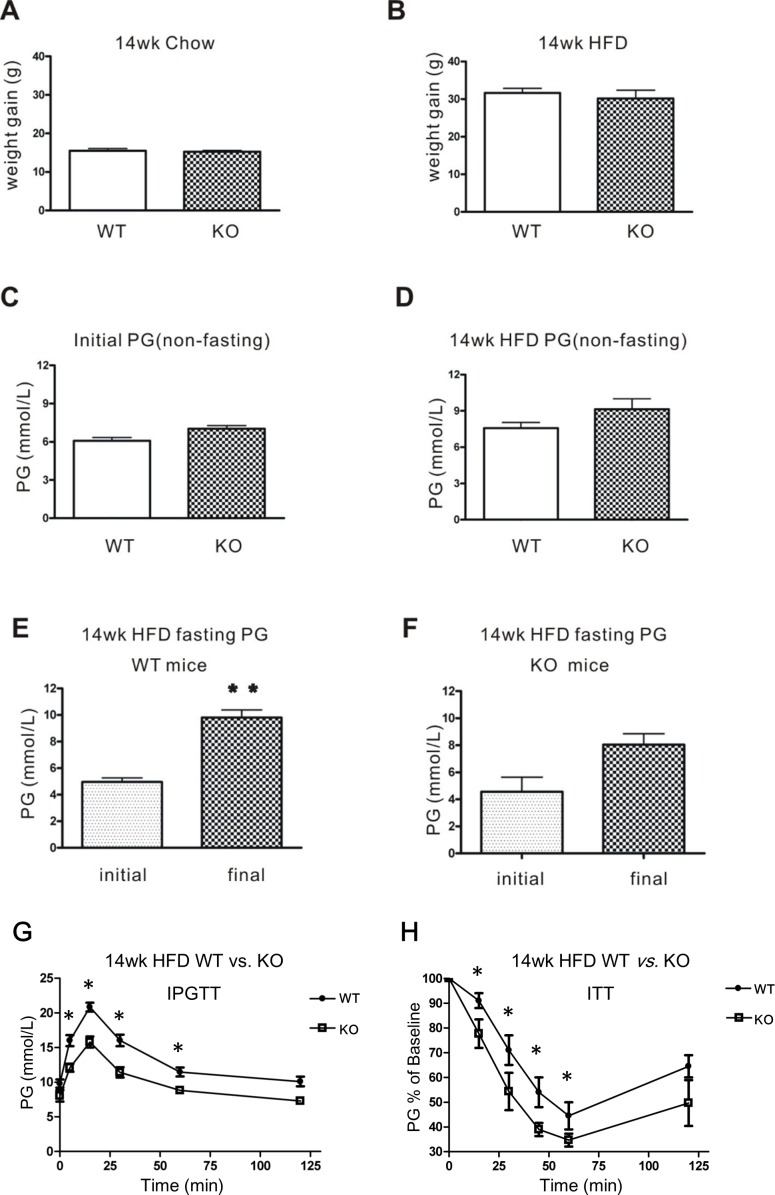
Glucose homeostasis in WT and CD226 KO mice after 14 weeks of high-fat diet (HFD) After 14 weeks of regular chow **A.** or high-fat diet **B.**, body weight gains were measured in WT and CD226 KO mice. Non-fasting PG did not differ between C57BL/6 WT and CD226 KO mice before **C.** or after **D.** high-fat diet (WT *vs*. KO, *p* > 0.05, *n* = 6-8). **E.** Fasting PG in WT mice (initial *vs.* final, **p < 0.01, *n* = 6-8), **F.** fasting PG was higher in CD226 KO mice than in WT mice, but this difference was only marginally significant (*p* = 0.06, *n* = 6-8). **G.** Intraperitoneal injections of glucose in fasting HFD-fed WT (solid circles) and CD226 KO (hollow squares) mice led to lower glucose concentrations after 5, 15, 30, and 60 min. (*p < 0.05, **p < 0.01, *n* = 6-8). (H) The response of fasting HFD-fed WT (solid circles) and CD226 KO (hollow squares) mice following a single intraperitoneal injection of insulin (1.5 U/kg) was monitored by repeated blood glucose concentration measurements. Glucose concentrations were reduced more in HFD-fed CD226 KO mice than in HFD-fed WT mice after 15, 30, 45, 60 min (*p < 0.05, *n* = 6-8).

Next, CD226 KO mice and C57BL/6 WT mice were fed a HFD over 14 weeks. No differences in body weight gain or non-fasting plasma glucose (PG) were observed between CD226 KO mice and WT animals fed with normal chow or HFD (Figure 5A-5D). However, after 14 weeks of HFD, glucose metabolic capacity decreased in WT mice (*p* < 0.01); there was also a trend towards a decrease in CD226 KO mice (Figure 5E and 5F). However, CD226 KO mice performed better in an Intraperitoneal glucose tolerance test (IPGTT) after 14 weeks of HFD (Figure 5G). Similarly, HFD CD226 KO mice were more sensitive to insulin than HFD WT animals as measured by an insulin tolerance test (ITT) (Figure 5H).

**Figure 6:**
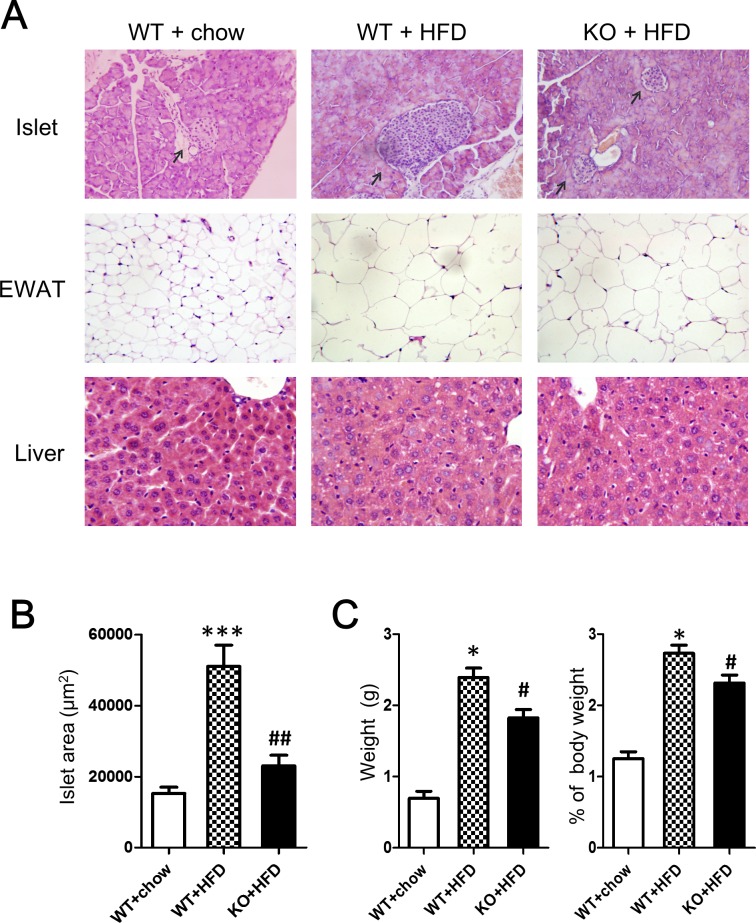
Histopathological alterations were evaluated using HE staining **A.** Representative images of pancreatic islets, EWAT, and livers of mice fed normal (chow) or HFD for 14 consecutive weeks. (Pancreatic islets and EWAT images are displayed at ×200 original magnification, livers are displayed at ×400 original magnification). *N* = 3-5 for each group of mice. **B.** Islet area in μm^2^. Values are presented as the mean ± SEM of 6 observations per mouse, *n* = 3-5 mice per group. ***p < 0.001 compared to similar mice fed normal diet. ^##^*p* < 0.01 compared to WT C57BL/6 mice fed same diet. **C.** Epididymal white fat pads excised from HFD-fed WT and CD226KO mice were weighed after removing non-white-adipose tissues. Absolute (left) and relative values (right) are shown. *p < 0.01 compared to WT mice fed normal diet. ^#^*p* < 0.05 compared to WT C57BL/6 mice fed HFD.

Representative hematoxylin-eosin (HE) images showing pancreatic islets, livers, and adipose tissue in mice given normal diets or HFD are shown in Figure 6. Compared with normal diet controls, islet size was increased in HFD-fed WT C57CL/6 mice (*p* < 0.001), indicating alterations in beta cells to slow progression to hyperglycemia. Notably, HFD reduced the increase in islet size in CD226 KO mice compared to WT C57CL/6 mice (*p* < 0.01), suggesting that CD226 deletion may slow the development of insulin resistance. Histological analysis showed severe microvesicular steatosis in the liver and adipocyte hypertrophy in epididymal white adipose tissue (EWAT) in both WT C57BL/6 and CD226 KO HFD-fed mice. There were no differences in macrophage infiltration into EWAT between HDF-fed WT and CD226 KO mice (Supplemental Figure 2).

### Glucose challenge increased soluble CD226 levels in T2DM patients

**Figure 7:**
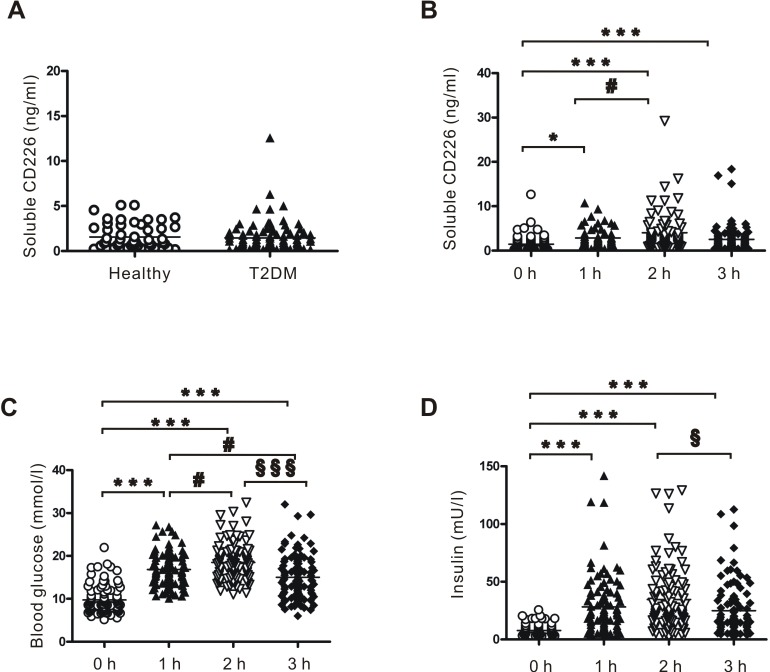
Plasma levels of soluble CD226 detected by ELISA **A.** Fasting plasma levels of soluble CD226 in healthy controls (*n* = 50, hollow circles) and T2DM (*n* = 94, solid triangles) patients. **B.** Plasma levels of soluble CD226 from T2DM patients before (0 h, hollow circles) and 1 h (solid triangles), 2 h (inverted hollow triangles) and 3 h (solid diamonds) after an oral glucose tolerance test (OGTT). Results of the OGTT with time courses of blood glucose **C.** and insulin **D.** concentrations. Median values and interquartile ranges are shown. ***p < 0.001 *vs.* 0 h; ^#^*p* < 0.05 *vs.* 1 h; ^§^*p* < 0.05 *vs*. 2h; §§§p < 0.001 *vs*. 2 h.

Many transmembrane receptors are released from the cell surface into circulation in soluble forms during activation. Using sandwich ELISA, we found that soluble CD226 levels in fasting plasma were indistinguishable between T2DM patients (*n* = 94, range: 0.10-12.57 ng/ml, median: 1.45 ng/ml) and healthy controls (*n* = 50, range: 0.10-5.05 ng/ml, median: 1.57 ng/ml) (Figure 7A).

Interestingly, soluble CD226 plasma concentrations in T2DM patients were higher 1 h after an oral glucose tolerance test (OGTT) (range: 0.10-65.90 ng/ml, median: 2.84 ng/ml) than during fasting (0 h) (*p* < 0.01). Furthermore, soluble CD226 concentrations remained elevated 2 h (range: 0.34-58.70 ng/ml, median: 4.06 ng/ml) and 3 h (range: 0.24-18.39 ng/ml, median: 2.54 ng/ml) post-OGTT compared to fasting levels (*p* < 0.001) (Figure 7B). Changes in blood glucose and insulin levels during OGTT were also measured at the same time points (Figure 7C and 7D, respectively). Increased endothelial cell CD226 expression in diabetic patients may increase CD266 shedding from the cell membrane, ultimately increasing soluble plasma levels as well.

Plasma levels of soluble CD226, blood glucose, and insulin after OGTT were also measured in healthy controls (*n* = 10). There were no differences in soluble CD226 at any of the post-OGTT time points compared to during fasting (Supplemental Figure 3). Glucose and insulin levels after OGTT in healthy controls are shown in Supplemental Figure 3.

## DISCUSSION

In this study, we discovered a new role for CD226 in metabolic homeostasis. Although CD226 KO mice on a HFD developed obesity, they were partially protected from the development of glucose intolerance. T2DM is associated with increased endothelial dysfunction and atherosclerotic vascular diseases. Hyperglycemia may stimulate the expression of adhesion molecules on endothelial cells and increase the transendothelial migration (TEM) of monocytes [15]. Substances that inhibit high glucose-induced adhesion molecule expression in endothelial cells, such as flavonoid apigenin and selenium, may reduce vascular damage [16, 17]. CD226 is a known inducible adhesion molecule on human endothelial cells [18].

Little is known about the regulation of glucose uptake in endothelial cells. Both GLUTs and SGLT-1 are present in endothelial cells and intact coronary artery endothelium and likely contribute to glucose uptake. For example, insulin acts through GLUT1/4 translocation to produce rapid glucose uptake [19-21], and insulin increased endothelial cell glucose uptake in the present study.

Transcellular glucose transport in endothelium may contribute to the movement of glucose from the blood to the vascular wall [21]. Treatment of endothelial cells and monocytes with antibodies against CD226 and CD155 arrests monocyte and effector memory T cell transport across the endothelial junction and prevents TEM [22-24]. It is also possible that combined inflammatory and hyperglycemic states increase CD226 levels, which inhibits transendothelial glucose transport.

The cytoskeleton is essential for maintaining endothelial integrity. Previous studies demonstrate that TNF-α increases endothelial cell permeability in a dose- and time-dependent manner and exerts a toxic effect [25]. Our data demonstrate that CD226 knockdown protects against TNF-α-induced stress fiber formation and cytoskeleton remodeling, indicating that inflammation-induced CD226 increases may aggravate endothelial dysfunction in diabetic hyperglycemia.

Generally, soluble adhesion molecules block the binding of ligands to membrane receptors. In many cases, circulating soluble receptors are also used as biomarkers of disease severity; for example, increases in soluble CD163 levels are found in high-risk T2DM individuals [26, 27]. Increases in soluble CD226 (sCD226) have been detected in many diseases and clinical disorders. Elevated sCD226 in HIV and cancer patients suggests that CD226 shedding from cell membranes occurs in a variety of diseases and could potentially be used in therapeutic monitoring [28, 29]. In this study, we found no differences in sCD226 levels between healthy individuals and T2DM patients. However, sCD226 levels in T2DM at 1, 2, and 3 h after OGTT were significantly higher than fasting levels. This increase in sCD226 may reflect increased membrane expression of CD226 under the inflammatory conditions found in diabetic patients.

Although CD226 deficiency did not protect mice from HFD-induced weight gain, it reduced glucose intolerance, as shown by IPGTT and ITT. Similarly, HFD evoked a compensatory increase in islet size in WT mice, but not in CD226KO mice. Notably, CD226 levels increased under inflammation and high glucose conditions, while CD226 knockdown increased endothelial cell glucose uptake. Furthermore, sCD226 levels increased rapidly following OGTT in T2DM patients, but not healthy individuals. Further experiments are needed to define the molecular mechanism by which CD226 reduces glucose uptake in endothelial cells.

## MATERIALS AND METHODS

### RNA isolation and RT-PCR

Total RNA was extracted from cultured cells using TRIZOL Reagent according to the manufacturer's protocol (Invitrogen Life Technologies, USA). First-strand cDNA was synthesized in a volume of 20 μl using 1 μg of total RNA and the PrimeScript^®^ RT reagent (DRR036A). RNA was quantified at 260 nm in a spectrophotometer with an OD 260/280 ratio of 1.8 to 2.0. CD226 and β-actin mRNA expression were analyzed by RT-PCR. The primer pairs were as follows: CD226 (forward): 5′-CATAACTTAACCCAGGTGGAGTG-3′, CD226 (reverse): 5′-ATGTCTGCTTCTGAGGCATTGTA-3′; β-actin (forward): 5′-GACTACCTCATGAAGATC-3′, β-actin (reverse): 5′-GATCCACATCTGCTGGAA-3′. Two microliters of cDNA were amplified by PCR using 30 cycles of denaturation at 94°C for 20 s, annealing at 55°C or 30 s, and extension at 72°C for 20 s. After PCR, 10 μl aliquots were electrophoresed on 2% agarose gel and visualized using 0.2% ethidium bromide and ultraviolet transillumination.

### Hematoxylin-eosin and immunohistochemistry (IHC) staining

For histological analysis, tissues were fixed in 10% neutral-buffered formalin and embedded in paraffin. 4 μm thick sections were affixed to slides, deparaffinized, and stained with hematoxylin and eosin to determine morphological changes. Islet area was analyzed in a blinded fashion using Image-Pro Plus 6.0 software (Media Cybernetics, Silver Spring, USA) and expressed as μm^2^.

For IHC staining, sections were treated for 10 min in 0.3% H_2_O_2_/methanol to block endogenous peroxidase activity. After three PBS washes, sections were incubated with 10% normal goat serum to reduce non-specific protein binding and incubated overnight at 4°C with rabbit anti-mouse CD226 polyclonal antibody. Primary antibodies were detected using a streptavidin-horseradish peroxidase (HRP) kit (Immunocruz staining system, Santa Cruz, Dallas, TX, USA) according to the manufacturer's instructions. The antibody complexes were then visualized by incubation with 3,3′-diaminobenzidine (DAB) chromogen. The sections were counterstained with Mayer's hematoxylin for 30 s, dehydrated through an ethanol series, cleared, mounted, and examined using light microscopy.

To observe the infiltration of macrophages into EWAT, sections were blocked with 10% normal goat serum and incubated overnight at 4°C with rat anti-mouse F4/80 monoclonal antibody (Abcam, CI:A3-1, Cambridge, England). After 3 washes, the primary antibodies were detected using a Cy3 goat anti-rat secondary antibody. Cell nuclei were stained with Hoechst 33342. Images were captured by a Zeiss LSM 800 confocal microscope and analyzed using ZEN lite software.

### Cell isolation and culture

HUVECs obtained from umbilical cords were digested with 0.05% type II collagenase (Sigma Chemical Co., St. Louis, MO, USA). Isolated HUVECs were cultured in 20% fetal bovine serum (FBS) (Gibco, Grand Island, USA) in Clonetics TM endothelial cell systems (Lonza, Walkersville, USA) until confluent. Passages 3-5 were starved with 2% FBS in culture medium for 16-24 h before the experiments. For Western blot analysis, HUVECs were divided into two groups: the normal glucose group (cells were cultured in EGM-2 medium containing 5.5 mM glucose), and the high glucose group (glucose was added to the medium for a final concentration of 30 mM). Then, cells were stimulated with or without 200 μM palmitate, 10 ng/ml human TNF-α, or 200 μM CoCl_2_ (Sigma) for 12 h. Then, HUVECs on dishes were washed with PBS, scraped with lysis buffer, incubated on ice for at least 30 min, and centrifuged at 14,000 rpm for 30 min. The supernatants were stored as cellular lysates at −80°C before analysis.

### Western blotting

HUVECs were grown to confluency in 35 mm dishes or 6-well plates. Total cellular proteins were extracted in RIPA buffer. Membrane proteins were isolated using the “Membrane and Cytosol Protein Extraction Kit” (Beyotime LLC, China). Cellular proteins were quantified with a BCA Protein Assay Kit (Pierce, Rockford, USA). Samples were loaded onto an SDS-PAGE gel and proteins were then transferred onto PVDF membrane for Western blot analysis. Blots were blocked with 5% dry milk in PBS with 0.1% Tween-20 for 1 h at room temperature and then incubated with rabbit anti-mouse/human CD226 (Sigma-Aldrich, Saint Louis, MO, USA) or rabbit anti-Glut1 polyclonal (Novus Biologicals, LL, USA) primary antibody overnight at 4°C. After washing, HRP-labled secondary antibodies (Pierce Biotechnology, Inc., Rockford, IL, USA) were added at a dilution of 1:5000 for 1 h at room temperature. The blots were visualized with ECL-Plus reagent (GE Healthcare, Piscataway, NJ). GAPDH antibody was used to confirm equal protein loading.

### Transduction of lentivirus

For CD226 knockdown, lentivirus encoding CD226 shRNA (5′-GCACTGTGTGAAGAGACATTG-3′) or scrambled control shRNA (5′-ACTATATATCGTCCTTAAGCT-3′) was purchased from Genechem (Shanghai, China). The shRNA sequence was cloned into a GV118 lentiviral vector with the human U6 promoter. HUVECs were seeded into 6-well plates for 24 h until 80% confluent and infected with lentivirus with a multiplicity of infection (MOI) of 100 plaque-forming units (pfu) per cell. After incubation with viral particles for 48 h, cells were assessed for transduced gene expression. 10 ng/ml human TNF-α was added 12 h before the glucose uptake assay.

### Glucose uptake assay

HUVECs were plated in 24-well plates and cultured in high glucose culture medium with 20% FBS. Cells were treated with vehicle, 10 ng/ml recombinant human TNF-α, or TNF-α plus CD226 RNAi lentivirus for 2 d. Control group cells were pre-incubated with 10^−7^M insulin for 2 h before the D-glucose analog 2-[N-(7-nitrobenz-2-oxa-1,3-diazol-4-yl) mino]-2-deoxy-d-glucose (2-NBDG) was added. For experiments, all culture medium was removed from each well and replaced with 500 μl of fresh, high glucose DMEM in the absence or presence of fluorescent 2-NBDG (100 μM). Plates were incubated at 37°C with 5% CO_2_ for 30 min as described previously [30]. 2-NBDG uptake was stopped by removing the incubation medium and washing the cells twice with pre-chilled PBS. Cells in each well were subsequently digested, suspended and maintained at 4°C for flow cytometry analysis performed within 30 min. Cell events were collected using FACS Calibur and FACS C6 flow cytometers (Becton Dickinson Immunocytometry Systems, SanJose, CA) and analyzed by FlowJo 7.6 software (FlowJo, LLC, Ashland, OR, USA).

### RT^2^ Profiler^TM^ PCR array

Total RNA was extracted from the livers of HFD-fed WT or CD226 KO mice using TRIZOL Reagent and the RNeasy MinElute Cleanup Kit (Qiagen, Germany). SuperScript Reverse Transcriptase was applied to reverse-transcribe first-strand cDNA. To analyze the differential expression of multiple genes involved in diabetes mellitus, we used the Mouse Glucose Metabolism RT^2^ Profiler^TM^ PCR Array (PAMM-006A, SA Bioscience, Qiagen), which uses SYBR Green-based real-time PCR to assay a large number of genes simultaneously. Each array contained 84 specific cDNA fragments of genes involved in glucose metabolism, 5 housekeeping genes to normalize array data (HK1-5), 3 wells containing replicate reverse-transcription controls (RTC), 3 wells containing replicate positive PCR controls (PPC), and a well containing a genomic DNA control (GDC). Supplemental Table 1 lists the genes measured. We added cDNA to each well of an RT^2^ profiler PCR array for quantitative PCR in the Bio-Rad system with the following cycling conditions: an initial denaturation at 95°C for 15 minutes followed by 40 cycles of 95°C for 15 seconds and 55°C for 60 seconds, with a final infinite 4°C hold. Each replicate cycle threshold (Ct) was normalized to the average Ct of 5 endogenous controls on a per plate basis. The comparative Ct method was used to calculate relative gene expression.

### Phalloidin staining

HUVECs were grown on glass chamber slides and treated with 30 mM (high) glucose for 12 h with or without 10 ng/ml of human TNF-α. The cells were fixed with 3.7% paraformaldehyde for 30 min, then washed with PBS and permeabilized with 0.1% Triton X-100 in PBS for 5 min. The cells were then washed, incubated for 30min at 37°C with FITC-labeled phalloidin (Molecular Probes, USA) and viewed with a confocal microscope (Olympus, Japan). The images were visualized using FLUOVIEW ver.1.4a viewer software.

### Animals

Male C57BL/6 mice (4-5 weeks of age) were purchased from the animal center of the Fourth Military Medical University (Xi'an, China). CD226 KO mice from a C57BL/6 background were kindly provided by Prof. Marco Colonna (Department of Pathology and Immunology, Washington University School of Medicine). Genotypes of offspring were determined by PCR analysis of tail-extracted DNA. Mice were housed and maintained in clean cages and fed with standard chow and water before HFD. The mice were separated into four groups with similar mean body weights. WT C57BL/6 mice were fed with a regular chow diet (chow, Medicience Ltd #MD12031: 10% of calories derived from fat) or a high fat diet (HFD, Medicience Ltd #MD12032: 45% of calories derived from fat) for 14 weeks. All experiments using mice were approved by the Fourth Military Medical University Animal Experimental Committee.

### Intraperitoneal glucose tolerance test (IPGTT) and insulin tolerance test (ITT)

Plasma glucose (PG) in whole blood obtained from mouse tail veins (0 min) was measured by a glucometer (Life-Scan, Milpitas, CA) after 12 h of fasting. Later, glucose solution was injected intraperitoneally (1 g/kg body weight) and plasma glucose was assayed after 5, 15, 30, 60, and 120 min. ITT was carried out in animals that were fasted for 6 hours. An intraperitoneal bolus injection of recombinant human regular insulin (1.5 U/kg) (Novolin R; Novo Nordisk Inc.) was administered; blood glucose concentrations were measured using a glucometer before (0 min) and 15, 30, 45, 60, and 120 min after injection.

### Patients and controls

For the detection of soluble CD226 during fasting, plasma samples from patients with T2DM were randomly selected from the clinical laboratory of Xijing Hospital of Fourth Military Medical University (*n* = 94, 34 female and 60 male). All patients were diagnosed with T2DM according to American Diabetes Association guidelines [31] (mean duration of diabetes = 8.2 years; mean age = 50 years). Subjects with inflammatory disorders, microvascular and macrovascular complications, abnormal liver, renal, or thyroid function, or steroid therapy or anti-inflammatory drug use were excluded. 50 healthy control subjects (19 female and 31 male; mean age = 30 years) with normal complete blood count, no family history of diabetes or other chronic diseases, and fasting plasma glucose (FPG) levels < 100 mg/dL were included.

For the detection of soluble CD226 in the oral glucose tolerance test (OGTT), plasma samples from these 94 T2DM patients and 10 healthy subjects (6 female and 4 male; mean age = 27 years) were examined. FPG, 1-h plasma glucose (1-h PG), 2-h PG, and 3-h PG levels in the OGTT were assayed using standard laboratory techniques. All procedures were approved by the Xijing Hospital ethics committee, and all participants provided written informed consent. After measuring the clinical parameters, plasma samples were aliquoted and stored at −20°C until use.

### Sandwich ELISA

The anti-human CD226 monoclonal antibodies (mAbs) and ELISA kit were used as previously described [28]. Briefly, 100 μl of anti-human CD226 mAb (5 μg/ml in 0.05 M sodium carbonate buffer, pH 9.5) was added to each well of a Nunc Maxisorp ELISA plate (Nunc, Rochester, NY, USA) and incubated overnight at 4°C. After a 3x wash, plasma samples or standard CD226 recombinant protein serially diluted with PBS containing 0.1% BSA and 0.1% Tween-20 were added to the wells and incubated for 1 h at 37°C. After extensive washing with PBS containing 0.1% Tween-20 (PBST), the wells were incubated with another anti-human CD226 mAb conjugated with biotin for 1 h at 37°C. Then, 100 μl of commercial streptavidin-horseradish peroxidase (HRP) was added and color development was performed on a TMB visualized system. 450 nm absorbance was determined with a microplate reader (Bio-Rad, Hercules, CA, USA).

### Statistical analysis

Results are expressed as mean ± SEM. Statistical analysis was performed using Student's *t*-test or ANOVA where appropriate. For the patients' data, distribution was first determined using the Kolmogorov-Smirnov test. Results are reported as median ± range for nonparametric data (non-normal distribution). Differences were analyzed using the Mann-Whitney test for two groups and the Kruskal-Wallis test for three or more groups with nonparametric data. All statistical tests were performed with GraphPad Prism software (GraphPad Software, San Diego, CA). A *p* value < 0.05 was considered statistically significant.

## SUPPLEMENTARY MATERIAL TABLE AND FIGURES


