# Supplementary material for: CD226 reduces endothelial cell glucose uptake under hyperglycemic conditions with inflammation in type 2 diabetes mellitus
Source: Oncotarget. 2016 Feb 19;7(11):12010–23. doi: 10.18632/oncotarget.7505 (PMC4914265; doi:10.18632/oncotarget.7505)
Supplement: Supplementary file 1 [file oncotarget-07-12010-s001.pdf]

CD226 reduces endothelial cell glucose uptake under hyperglycemic conditions with inflammation in type 2 diabetes mellitus

Supplementary Material

Supp Table 1. Gene list of RT^2 profile^{TM} PCR mouse glucose metabolism arrays

Pdp2	Pdpr	Acly	Aco1	Aco2	Agl	Aldoa	Aldob	Aldoc	Bpgm	Cs	Dlat
Dld	Dlst	Eno1	Eno2	Eno3	Fbp1	Fbp2	Fh1	G6pc	G6pc3	G6pdx	Galm
Gapdhs	Gbe1	Gck	Gpil	Gsk3a	G3k3b	Gys1	Gys2	H6pd	Hk2	Hk3	Idh1
Idh2	Idh3a	Ish3b	Idh3g	Mdh1	Mdh1b	Mdh2	Ogdh	Pck1	Pck2	Pcx	Pdha1
Pdhb	Pdk1	Pdk2	Pdk3	Pdk4	Pfk1	Pgam2	Pgk1	Pgk2	Pgm1	Pgm2	Pgm3
Phka1	Phkb	Phkg1	Phkg2	Pklr	Prps1	Prps1l1	Prps2	Pygl	Pygm	Rbks	Rpe
Rpia	Sdha	Sdhb	Sdhc	Sdhd	Sucla2	Suclg1	Suclg2	Taldo1	Tkt	Tpil	Ugp2
Gusb	Hprt	Hsp90ab1	Gapdh	Actb	MGDC	RTC	RTC	RTC	PPC	PPC	PPC

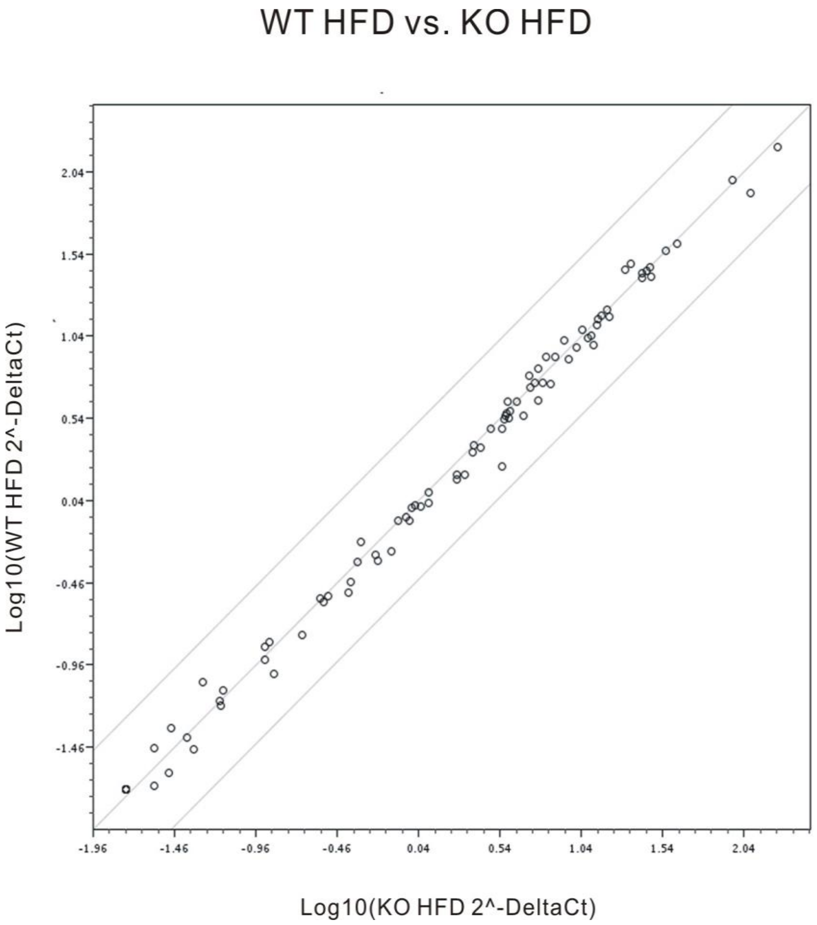

Supplemental figure 1: Genes related to glucose metabolism were analyzed using the RT^2 Profiler^{TM} PCR Array. The liver tissues were obtained from WT and CD226 KO mice fed with HFD for 14 weeks. Data was analyzed using SABiosciences web-based software.

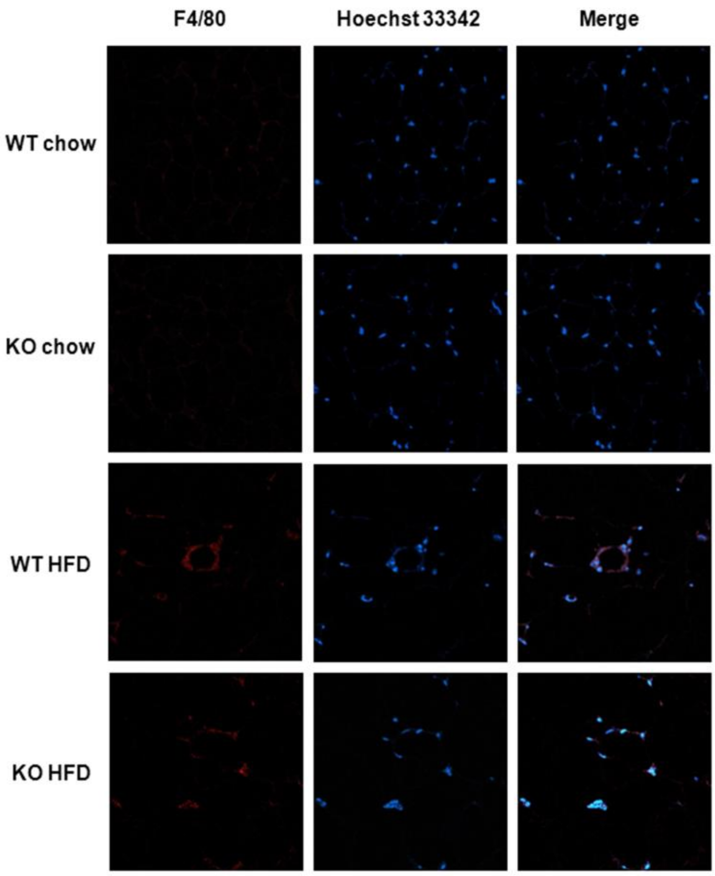

Supplemental figure 2: Immunofluorescence staining and confocal detection of F4/80-positive macrophages (red) in epididymal fat tissue of WT and CD226 KO mice fed chow or HFD for 14 wks. Nuclei were stained with Hoechst 33342 (blue). Representative images are displayed at 200× original magnification, n = 3-5 for each group of mice.

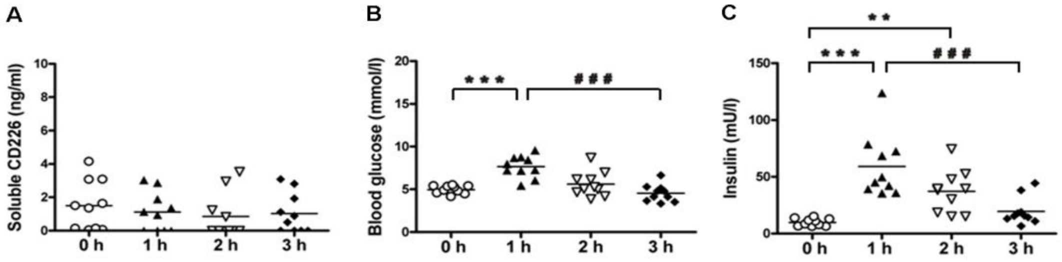

Supplemental figure 3: (A) Plasma levels of soluble CD226 from healthy subjects (n = 10) before OGTT (0 h, hollow circles), or 1 h (solid triangles), 2 h (inverted hollow triangles) and 3 h (solid diamonds) after OGTT. Results of the OGTT with time courses of blood glucose (B) and insulin (C) concentrations. Median values and interquartile ranges are shown. $^{***}p < 0.001$ vs. 0 h; $^{**}p < 0.01$ vs. 0 h; $^{###}p < 0.001$ vs. 1 h.
